# Identifying the Conditions Under Which Antibodies Protect Against Infection by Equine Infectious Anemia Virus

**DOI:** 10.3390/vaccines2020397

**Published:** 2014-05-27

**Authors:** Elissa J. Schwartz, Robert J. Smith?

**Affiliations:** 1 School of Biological Sciences, Department of Mathematics, Washington State University, Pullman, WA 99164, USA; E-Mail: ejs@wsu.edu; 2 Department of Mathematics and Faculty of Medicine, University of Ottawa, Ottawa, ON K1N 6N5, Canada

**Keywords:** equine infectious anemia virus, vaccination, antibodies, mathematical modeling, lentivirus

## Abstract

The ability to predict the conditions under which antibodies protect against viral infection would transform our approach to vaccine development. A more complete understanding is needed of antibody protection against lentivirus infection, as well as the role of mutation in resistance to an antibody vaccine. Recently, an example of antibody-mediated vaccine protection has been shown via passive transfer of neutralizing antibodies before equine infectious anemia virus (EIAV) infection of horses with severe combined immunodeficiency (SCID). Viral dynamic modeling of antibody protection from EIAV infection in SCID horses may lead to insights into the mechanisms of control of infection by antibody vaccination. In this work, such a model is constructed in conjunction with data from EIAV infection of SCID horses to gain insights into multiple strain competition in the presence of antibody control. Conditions are determined under which wild-type infection is eradicated with the antibody vaccine. In addition, a three-strain competition model is considered in which a second mutant strain may coexist with the first mutant strain. The conditions that permit viral escape by the mutant strains are determined, as are the effects of variation in the model parameters. This work extends the current understanding of competition and antibody control in lentiviral infection, which may provide insights into the development of vaccines that stimulate the immune system to control infection effectively.

## 1. Introduction

Despite advances in our understanding of the control of viral infection, a vaccine is still needed to best control human immunodeficiency virus type 1 (HIV-1) and other viruses that result in chronic infections. Knowledge of how antibodies can block the establishment of initial infection would transform our approach to vaccine development. However, the antiviral effectiveness of the initial antibody response is under debate [1,2]. What is needed is the ability to predict the conditions under which antibodies could protect against infection.

Equine infectious anemia virus (EIAV) is a macrophage-tropic lentivirus that establishes a chronic, persistent viral infection in horses and ponies [3,4]. Infected animals are typically able to control the viral infection throughout their lifetimes, with control mediated by antibody and cellular immune responses [5,6]. EIAV infection is used as an experimental system for the study of the immune control of persistent infection [7]. As such, it is valuable for research focused on the development of protective vaccines against EIAV and related lentiviruses, including HIV-1 [8,9].

Horses with severe combined immunodeficiency (SCID) serve as a useful tool to examine viral dynamics in animals without adaptive immune responses. Several recent studies [10,11] describe protection from EIAV infection due to passively transferred neutralizing antibodies in horses with SCID. SCID is a naturally occurring condition in which horses lack the ability to make adaptive immune responses, including B-cells and T-cells; therefore, these horses do not produce antibodies or cytotoxic T lymphocytes (CTLs). Infusion of SCID foals with plasma from a long-term EIAV-infected immunocompetent horse conferred upon them EIAV-specific neutralizing antibodies, which protected them from wild-type EIAV infection [10,11]. Passive antibody transfer has also shown that neutralizing antibodies can block infection with chimeric simian/human immunodeficiency virus (SHIV) in rhesus macaques [12,13,14,15,16].

Horses were given three infusions of plasma that contained broadly neutralizing antibodies to a number of EIAV strains on Days −1, 7 and 14, with EIAV challenge occurring on Day 0 [10,11]. While the passive transfer of convalescent immune plasma protected the horses from wild-type infection, a mutant strain was seen to emerge after approximately five to seven weeks in several horses. This mutant was found to exist in the inoculum at a low level. These experiments show the plausibility of a scenario in which antibodies neutralize a wild-type virus strain. This strain does not persist, even though antibody levels decay and do not regenerate in the horse, except due to subsequent infusions. The wild-type strain is eliminated, but a neutralization-resistant mutant strain is selected and grows. This example provides a unique opportunity to learn about the control of lentiviral infection by antibody vaccination, as well as about competition between wild-type and mutant strains under such a scenario.

Mathematical modeling of the interactions between viruses and immune system components can be a useful tool to understand the correlates of infection control. Particularly, modeling neutralizing antibody protection from EIAV infection in SCID horses may lead to insights into the mechanisms of control of infection by antibody vaccination. Previous modeling of EIAV derived thresholds for determining immune responses to successfully control infections [17] and analyzed virus–infected cell dynamics with two viral strains and constant or decaying antibody levels [18]. Modeling has been used to investigate, for example, the vaccine frequency and strength needed to control the number of HIV-infected cells with repeated administrations of a CTL vaccine [19]. Another study followed up on this work to examine the effect of mutation on CTL vaccine resistance [20]. However, we have yet to understand virus control with finite doses of an antibody vaccine, as well as the role of mutation on resistance to the antibody vaccine. As suggested in a recent article [7], we hypothesize that there are three strains competing in this infection, where the persistence of some strains may depend on the existence of the other strains. Strains (or viral sub-populations) arise continually: mutation in the wild-type gives rise to the first mutant strain at one mutation rate and to the second mutant strain at another mutation rate. The sub-populations are further defined by different growth rates and different sensitivities to antibody neutralization. In Taylor *et al.* [10], both the wild-type and first mutant strains were found to exist in the inoculum.

An impulsive mathematical model, in conjunction with the data from studies of EIAV-infected SCID horses [10,11], can be used to model the dynamics of the three infusions of EIAV-specific neutralizing antibodies. This model, parameterized with relevant clinical data, can be used to predict under what conditions we achieve the eradication of the wild-type strain with a finite number of antibody infusions. From this, we can also estimate unknown infection parameters, such as the antibody neutralization rate and the basic reproductive number, *R*_0_, which will indicate the threshold below which the infection cannot sustain itself. The effect of mutation on the antibody vaccine is also unknown. Furthermore, since the parameter values are likely to vary within a range, it would be useful to determine the effect of variation of the parameters on *R*_0_.

Impulsive differential equations consist of a system of ordinary differential equations (ODEs), together with difference equations. Between impulses, which occur at times *t_k_*, the system is continuous, behaving as a system of ODEs. At the impulse points, there is an instantaneous change in state in some or all of the variables. This instantaneous change can occur when certain spatial, temporal or spatio-temporal conditions are met [21,22,23,24]. This is related to the use of pulse vaccinations [25], seasonal skipping in recurrent epidemics [26], antiretroviral drug treatment [27] and birth pulses in animals [28]. The current study aims to understand the role that neutralizing antibody vaccines can play in the control of lentivirus infection. Conditions are determined under which wild-type infection is eradicated with the antibody vaccine. The conditions that permit viral escape by the mutant strains are also delineated. Unknown infection parameters are determined, including viral growth rates, the carrying capacity and the rate at which antibody neutralizes virus. The effect of varying the effectiveness of antibody infusion and neutralization is explored, as is the role of stochasticity in *R*_0_. Finally, conditions are derived whereby the presence of a highly mutable, but low-replicating, second mutant may in fact result in the persistence of the first mutant, to the exclusion of other strains. This work contributes to the understanding of virus control and potentially provides insights into the development of vaccines that stimulate the immune system to control infection.

## 2. Methods

We use ordinary differential equations to model three strains of the virus and impulsive differential equations to model the behavior of neutralizing antibodies. In the absence of vaccination, we assume that antibodies decay at rate *q* or are absorbed by the wild-type virus, Mutant 1 or Mutant 2 at rates *p_W_*, *p_M_*_1_ and *p_M_*_2_, respectively. The effect of vaccinating at times *t_k_* (*k* = −1, 7, 14) is to increase the antibody level by a fixed amount, *A^i^*.

We assume virus growth is logistic, using a form similar to a standard model in the ecological literature for population dynamics with logistic growth and harvesting [29,30]. This modeling formulation is preferable to modeling virus production at a constant rate with a linear death term, because our aim is to match the viral dynamics seen in EIAV infection, including two important characteristics: (1) the virus has the potential to reach a steady state in the absence of antibodies; and (2) the virus can be eradicated in the presence of antibodies. The logistic term represents virus production by infected cells that are subject to target-cell limitation [31] or to a maximal rate of virus production.

We also assume that virus is removed in proportion to neutralizing antibodies at rates *p_W_*, *p_M_*_1_ and *p_M_*_2_, respectively, and that the wild-type mutates to each mutant strain at rates *ϵ*_1_ and *ϵ*_2_, respectively. We also assume that the wild-type has higher replication and is more susceptible to the antibody response than Mutant 1; similarly, we assume that Mutant 1 has higher replication and is more susceptible to the antibody response than Mutant 2. However, we do not necessarily assume that mutation of the first mutant is higher than that of the second mutant.

The model is then:

$$\begin{array}{l} V_{W}^{\prime }=-p_{W}V_{W}A+r_{W}V_{W}(1-\frac{V}{K})-\epsilon_{1}V_{W}-\epsilon_{2}V_{W}-d_{W}V_{W}\\ V_{M1}^{\prime }=-p_{M1}V_{M1}A+r_{M1}V_{M1}(1-\frac{V}{K})+\epsilon_{1}V_{W}-d_{M1}V_{M1}\\ V_{M2}^{\prime }=-p_{M2}V_{M2}A+r_{M2}V_{M2}(1-\frac{V}{K})+\epsilon_{2}V_{W}-d_{M2}V_{M2} \end{array}$$

*A’* = −*qA* − *p_W _V_W_A* − *p_M_*_1_*V_M_*_1_*A* − *p_M_*_2_*V_M_*_2_*A*　　　*t* ≠ *t_k_*

∆*A* = *A^i^*　　　*t* = *t_k_*

Here *V* = *V_W_* + *V_M_*_1_ + *V_M_*_2_. We assume the following: *p_W_* > *p_M_*_1_ > *p_M_*_2_, *r_W_* > *r_M_*_1_ > *r_M_*_2_.

The list of parameters and their values is given in Table 1. The antibodies on Day 0 are calculated from *A^i^* on Day −1, with exponential decay at rate *q*. The value of *q* is calculated from the half-life of horse IgG [32]. The half-life of virus due to antibody neutralization, *t*_1_*_/_*_2_, was estimated using the half-life of SIV in animals that were CD8-depleted [33,34,35]. The viral growth rate was calculated by fitting data from EIAV-infected SCID horses to the model with antibody neutralization set to zero with the viral clearance rate subtracted. The carrying capacity was also fit to the same data and then adjusted to account for growth and clearance effects. Note that each strain has a different carrying capacity. See Appendix A for the details of the calculated parameters.

**Table 1:** Parameter values.

Parameter	Definition	Value	Range	Units	Reference
*r_W_*	Virus growth rate for wild-type in the absence of antibodies	23.60	0–46	day *^−^*^1^	Calculated (Appendix A)
*r_M_*_1_	Virus growth rate for first mutant	23.23	0–46	day *^−^*^1^	Calculated (Appendix A)
*r_M_*_2_	Virus growth rate for second mutant	23.09	0–46	day *^−^*^1^	Assumed
*K*	Virus carrying capacity	1.14 × 10^8^	7.47 × 10^8^–3.82 × 108	virus ml *^−^*^1^	Calculated (Appendix A)
*p_W_*	Wild-type virus neutralization by antibody	1.462 × 10*^−^*^2^ × *m*	(1.21 × 10*^−^*^2^–2.67 × 10*^−^*^2^) × *m*	ml mg *^−^*^1^ day*^−^*^1^	Calculated (Appendix A)
*p_M_*_1_	Mutant 1 virus neutralization by antibody	(varied)	—	ml mg *^−^*^1^ day*^−^*^1^	Assumed
*p_M_*_2_	Mutant 2 virus neutralization by antibody	(varied)	—	ml mg *^−^*^1^ day*^−^*^1^	Assumed
*q*	Antibody decay rate	0.0315	0.0277–0.0365	day *^−^*^1^	[ 32]
*ϵ*_1_	Mutation rate from wild-type to first mutant (per base per cycle)	2.7 × 10*^−^*^5^	1 × 10*^−^*^5^–3.4 × 10*^−^*^5^	day *^−^*^1^	[ 36]
*ϵ*_2_	Mutation rate from wild-type to second mutant (per base per cycle)	2.7 × 10*^−^*^6^	2 × 10*^−^*^6^–2.7 × 10*^−^*^2^**	day *^−^*^1^	Assumed
*A*^*i*^	Amount of antibody infusion	38.4 × *m*	(25.6–51.2) × *m*	mg ml *^−^*^1^	[ 10 , 11 ]
*A*_0_	Antibody on Day 0	37.2	24.9–49.4	mg ml *^−^*^1^	Calculated (Appendix A)
*V_W_*(0)	Number of wild-type viral particles that initiated infection	224	175–350	virus ml^−1^	[ 10,37]
*V_M_*_1_(0)	Number of Mutant 1 viral particles that initiated infection	9	—	virus ml^−1^	[ 10]
*V_M_*_2_(0)	Number of Mutant 2 viral particles that initiated infection	1	—	virus ml^−1^	Assumed
*t*_1_*_/_*_2_	Half-life of virus due to antibody neutralization	1.3	0.7–1.8	day	[ 33–35]
*d_W _*, *d_M_*_1_, *d_M_*_2_	Viral clearance rate	23	9.1–36	day^−1^	[ 38]
*m*	Antibody magnification factor	{1, 10, 50}	—	—	—

Neutralizing antibody infusions of 1 L plasma contained 640 mg of IgG per kg of body weight in a 60 kg horse, resulting in the estimate of *A^i^*. We assume the infused antibodies act systemically. The value of *V_W_*(0) was calculated from horse plasma volume and injection inoculum (10^6^ TCID_50_ [10]) using a conversion factor between TCID_50_ and plaque-forming units (PFU) of 0.7 [37]. The range of *V_W_*(0) assumed a horse plasma volume at time of injection of 2–4 L, which is based on a horse weight of 40–80 kg [10]. The value of *V_M_*_1_(0) was calculated given that 1 out of 25 single amplicons sequenced from the inoculum showed the first mutant sequence [10]. A second mutant sequence was not identified experimentally; here, one particle per ml was assumed to exist initially.

## 3. Results

### 3.1. Theoretical Results

We analyzed the non-impulsive system (that is, the system without antibody vaccination) in Appendix B. By analyzing the non-impulsive system, we gain insight into the long-term outcome in the absence of vaccination, which corresponds to a series of disturbances in the system.

There are four equilibria: the disease-free equilibrium (a steady state with no infection), an equilibrium with Mutant 1 alone (a steady state where the first mutant has out-competed both the wild-type and the other mutant), an equilibrium with Mutant 2 alone (corresponding to the dominance of the second mutant) and a coexistence equilibrium, where all three viral strains coexist. In all cases, the number of antibodies at equilibrium is zero, since the antibodies are eventually cleared and not replenished in SCID horses (in the absence of impulsive vaccination). It should be noted that there is no equilibrium with only the wild type, since the presence of the wild type always results in mutation occurring.

We also calculated the basic reproduction number (Appendix B.3). This is a threshold condition that determines whether the disease will persist or be eliminated [39]. We determined that the disease will persist if $R_{0}=\frac{r_{W}}{\epsilon_{1}+\epsilon_{2}+d_{W}}>1$, which will occur for our sample parameter values. *R*_0_ is a composite, consisting of five threshold values (*R*_1_, *R*_2_, *R_E_*_1_, *R_E_*_2_, *R_E_*_3_) that are derived from bifurcation properties of the existence (or otherwise) of endemic equilibria.

Persistence may take several forms. The virus may persist in the form of the coexistence equilibrium, or potentially in some oscillatory form or chaos (although we did not observe these numerically). The Mutant 1 equilibrium can in fact persist if the mutation rate is sufficiently high; surprisingly, this can occur even if the mutation rate of the second mutant is high. In the latter case, a second mutant that arises easily (but that is not as fit as the first) can in fact result in the persistence of the first mutant. See Appendix C for details.

**Figure 1:**
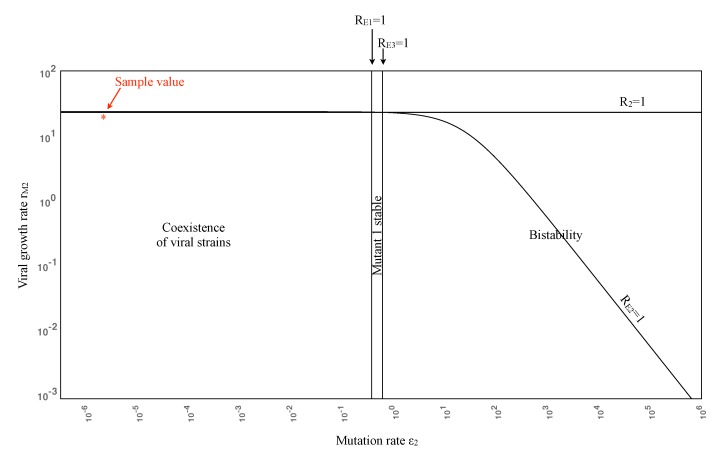
Viral persistence landscape diagram as Mutant 2 varies, showing that an increase in the mutation rate of Mutant 2 can stabilize the Mutant 1 equilibrium. The bistability region is included for completeness, but corresponds to unreasonably high mutation rates. Note the log scale on the axes.

Figure 1 illustrates the potential outcomes of viral strain persistence given the ranges of Mutant 2 growth and mutation rates. Note that *r*_*Mj*_ > *d*_*Mj*_ for *j* = 1, 2, so that the line *R*_2_ = 1 is an upper bound. Since the growth rate of Mutant 1 is assumed to exceed that of Mutant 2, it follows that the Mutant 2 equilibrium is always unstable (see Condition (1) in Appendix B). All parameters other than *r*_*M*2_ and *ϵ*_2_ are set to their sample values in Table 1, with *m* = 1. For $\epsilon_{2}<\frac{d_{M1}r_{W}}{r_{M1}}-\epsilon_{1}-d_{W}\approx 10^{-0.5}$, *R_E_*_1_ > 1 and *R_E_*_3_ > 1, so all three strains coexist. As *ϵ*_2_ increases, the Mutant 1 equilibrium becomes stable, so Mutant 1 persists. See Appendix B.1 for details.

As *ϵ*_2_ increases past 0.6 (corresponding to *R_E_*_3_ = 1), we move into a region of bistability: *R_E_*_3_*<* 1 and *R_E_*_1_*<* 1, so both the disease-free and Mutant 1 equilibria are stable. Bistability means that two equilibria are stable, so the ultimate result depends on the choice of initial conditions. Solutions that start near the disease-free equilibrium will approach it, while solutions that start near the Mutant 1 equilibrium will approach this equilibrium. However, this case is only included for completeness, since we expect that the mutation rate will not be this high in reality. Note also that the curve *R_E_*_2_ = 1 plays no role in the bifurcation, since Condition (1) holds.

We aimed to determine whether, according to the model, a finite number of impulses (*i.e.*, antibody infusions) could lead to virus elimination. A finite number of impulsive effects cannot fundamentally alter the long-term stability properties of equilibria. However, if the viral load falls below the eradication threshold of one viral particle in the horse, then the virus will be eradicated. With 3000 ml of plasma in the horse (since the horses weighed approximately 60 kg at the time of the experiment and plasma volume is approximately 5 percent of body weight [18,40]), this corresponds to an eradication threshold of one particle per 3000 ml.

### 3.2. Numerical Simulations

Using the sample values in Table 1, we performed numerical simulations to examine the transient and long-term behavior of the system. To investigate the effect of antibody control, we simultaneously increased both the antibody infusion, *A^i^* (and *A*_0_), and the neutralization ability, *p_j_* (*j* = *W,M*1*, M*2), by a magnification factor, *m*, where *m* = 1, 10, 50. (Note that all three virus neutralization rates were multiplied by *m*, regardless of their relative effect.) The value *m* = 10 means antibodies are ten times greater when infused and are 10 times more effective at neutralizing the virus. The magnification factor thus accounts for theoretical improvements on the vaccine.

We also examined the relative effectiveness of viral neutralization of mutants using three scenarios: the neutralization rates for both mutants are identical to the neutralization rate of the wild-type virus; Mutant 1 has 10-fold resistance and Mutant 2 has 100-fold resistance; and (3) both mutants have 100-fold resistance. The results are summarized in Table 2.

**Table 2:** The outcomes from changing antibody infusion and relative effectiveness.

Relative effectiveness		Antibody magnification factor *m*			Figure
*p_M_*_1_	*p_M_*_2_	1	10	50	
*p_W_*	*p_W_*	Wild-type dominates (coexistence)	Eradication (wild-type last)	Eradication (exponentially fast)	3
0.1*p_W_*	0.01*p_W_*	Wild-type dominates (coexistence)	Mutant 2 escape (others eradicated)	Eradication (Mutant 2 last)	4
0.01*p_W_*	0.01*p_W_*	Wild-type dominates (coexistence)	Mutant 1 escape (Wild-type eradicated)	Mutant 1 escape or eradication	5

**Figure 2:**
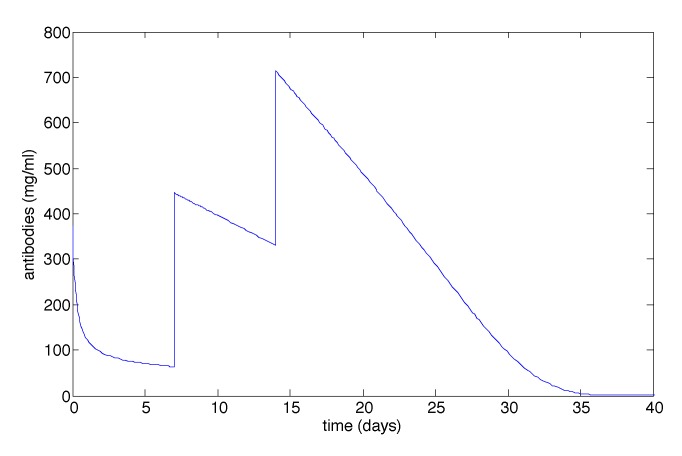
The antibody count for the case when both mutants have 100-fold resistance and *m* = 10. Antibody boosts occur on Day 7 and Day 14. This figure looks similar for other values of *m*.

Figure 2 illustrates the effect of the two antibody boosts (on Days 7 and 14) for the case when both mutants have 100-fold resistance and *m* = 10; see Table 2. An antibody boost on Day 7 has an instantaneous effect of increasing the antibody count. A final boost on Day 14 increases the antibody count again. After this time, the antibodies decay to zero after approximately 40 days.

**Figure 3:**
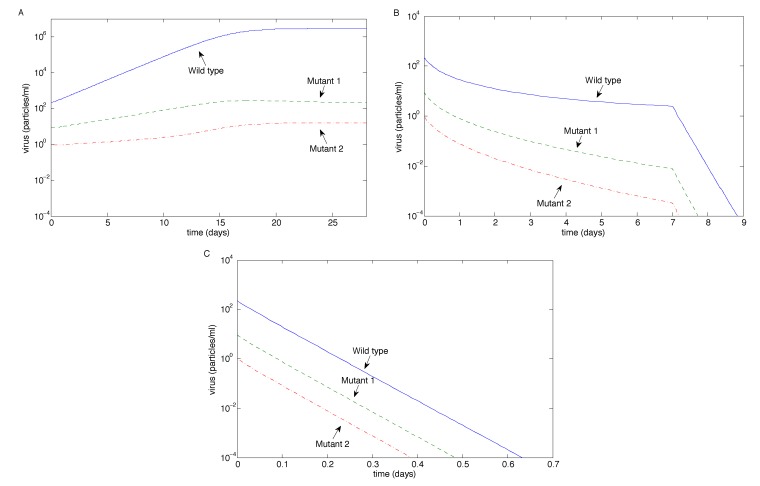
The long-term outcome for virus strains using the sample values in Table 1 for the case of equal virus neutralization rates (*p_M_*_1_ = *p_M_*_2_ = *p_W_*) as the antibody magnification factor *m* varies. (**A**) *m* = 1; (**B**) *m* = 10; (**C**) *m* = 50.

We first investigated the case when the antibody neutralization rates for all three strains are equal (*i.e.*, *p_M_*_1_ = *p_M_*_2_ = *p_W_*); see Figure 3. When antibody magnification is *m* = 1, all three strains coexist, but the wild-type dominates (note the log scale on the axes). Both 10-fold and 50-fold antibody magnifications eventually control all three strains of the virus. The sharp drop-off after seven days in the case of 10-fold antibody magnification corresponds to the first antibody boost on Day 7, which accelerates the eradication process. Eradication occurs exponentially quickly in the case of 50-fold magnification.

**Figure 4:**
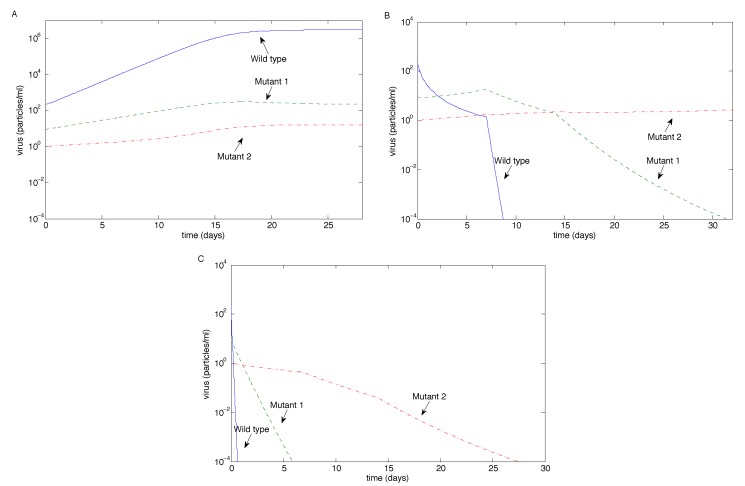
The long-term outcome using the sample values in Table 1 for the case when Mutant 1 has 10-fold resistance and Mutant 2 has 100-fold resistance (*p_M_*_1_ = 0.1*p_W_*, *p_M_*_2_ = 0.01*p_W_*) as the antibody magnification factor *m* varies. (**A**) *m* = 1; (**B**) *m* = 10; (**C**) *m* = 50.

Next, we examined the case when Mutant 1 had 10-fold resistance (*i.e.*, *p_M_*_1_ = 0.1*p_W_*) and Mutant 2 had 100-fold resistance (*i.e.*, *p_M_*_2_ = 0.01*p_W_*); see Figure 4. When antibody magnification is *m* = 1, all three strains coexist, but the wild-type dominates. Tenfold antibody magnification controls the wild-type and Mutant 1, but allows Mutant 2 to escape; note the accelerated decreases at Days 7 and 14 as antibodies are infused. However, 50-fold antibody magnification eventually controls all three strains of the virus; note that Mutant 2 is eradicated before the antibodies have decayed to zero.

**Figure 5:**
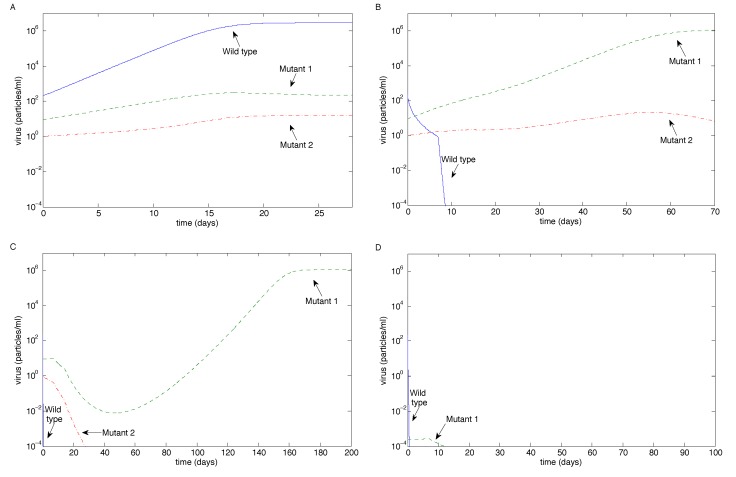
The long-term outcome using the sample values in Table 1 for the case when both mutants have 100-fold resistance (*p_M_*_1_ = 0.01*p_W_*, *p_M_*_2_ = 0.01*p_W_*) as the antibody magnification factor *m* varies. (**A**) *m* = 1; (**B**) *m* = 10; (**C**) *m* = 50; (**D**) the same as (C), except with no initial mutants.

We then examined the case when both mutants had 100-fold resistance (*i.e.*, *p_M_*_1_ = *p_M_*_2_ = 0.01*p_W_*); see Figure 5. When antibody magnification is *m* = 1, all three strains coexist, but the wild-type dominates. Tenfold antibody magnification controls the wild-type and reduces Mutant 2, but allows Mutant 1 to escape (reversing the outcome from the previous case); note that there are no antibodies after about 40 days, so Mutant 2 will eventually be out-competed by Mutant 1. Fifty-fold antibody magnification still allows Mutant 1 to escape, but controls Mutant 2; in this case, Mutant 1 is reduced, but not eradicated, when the antibodies decay to zero, allowing it to bounce back (Figure 5C). This is a different outcome from the two previous cases.

Due to the decay of Mutant 1, we further examined the case when there were no initial amounts of either mutant. Although Mutant 1 is seen experimentally [10], our aim was to examine the effect of initial-condition dependence. Figure 5D has identical parameters to Figure 5C, except that *V_M_*_1_(0) = *V_M_*_2_(0) = 0. In this case, Mutant 1 still emerges (due to the mutation rate, *ϵ*_1_), but quickly decays below the eradication threshold. We examined the issue of no initial mutants for all other cases, and the results were qualitatively unchanged in all figures (results not shown), except for Figure 5C. This suggests that initial fluctuations in Mutant 1 may affect the outcome when the antibody magnification rate is sufficiently high.

**Figure 6:**
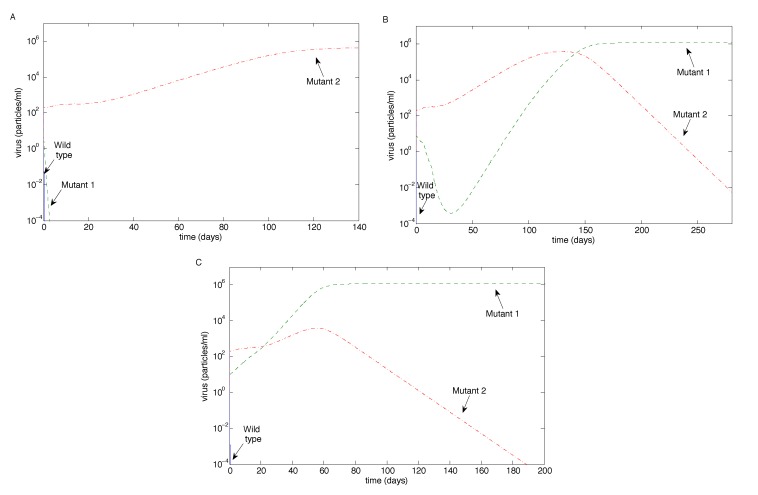
The case when the mutation rate of Mutant 2 is high (*ϵ*_2_ = 2.7 × 10^1^). Here, Mutant 1 has 10-fold resistance to the antibodies, Mutant 2 has 100-fold resistance to the antibodies and *m* = 10, recreating the conditions of Figure 3B, Figure 4B and Figure 5B (*i.e.*, the cases of 10-fold magnification), except for the high mutation rate of Mutant 2. (**A**) Unlike Figure 3B, Mutant 2 escapes; (**B**) unlike Figure 4B, Mutant 1 persists; conversely, Mutant 2 is eradicated, despite its extremely high mutation rate; (**C**) the persistence of Mutant 1, similar to Figure 5B.

We also examined the case when the mutation rate of Mutant 2 (*ϵ*_2_) was significantly higher than the mutation rate of Mutant 1 (*ϵ*_1_) for the case when Mutant 1 had 10-fold resistance and Mutant 2 had 100-fold resistance. Figure 6 is the analogue of Figure 3B, Figure 4B and Figure 5B (*i.e.*, when *m* = 10), but with a high mutation rate of Mutant 2. Figure 6A shows that Mutant 2 can escape if its mutation rate is sufficiently high, which is not surprising. Figure 6C is qualitatively unchanged from Figure 5B.

However, surprisingly, Figure 6B shows that Mutant 1 can persist, rebounding from low levels, while Mutant 2 is eradicated, despite having an extremely high mutation rate. In particular, while the mutation rate of Mutant 2 was high, Mutant 1 had a higher viral replication rate, although a lower resistance to antibodies. Mutant 2’s superior resistance to the antibodies allows it to dominate initially; however, when the antibodies decay to zero, Mutant 1’s superior replication allows it to out-compete Mutant 2 and hence dominate. We thus see the high mutation rate of Mutant 2 stabilizing the Mutant 1 equilibrium.

#### 3.2.1. Sensitivity Analysis

Since *R*_0_ determines whether the virus will persist or be eradicated, we are interested in the ability of variations in parameter values to affect *R*_0_. To examine this sensitivity of *R*_0_ to variations in parameters, we used Latin hypercube sampling and partial rank correlation coefficients (PRCCs) with 1000 Monte Carlo simulations per run. Latin hypercube sampling is a statistical sampling method that allows for an efficient analysis of parameter variations across simultaneous uncertainty ranges in each parameter by using repeated Monte Carlo simulations [41]. PRCCs illustrate the degree of the effect that each parameter has on the outcome. Parameters with positive PRCCs will increase *R*_0_ when they are increased, whereas parameters with negative PRCCs will decrease *R*_0_ when they are increased. However, the magnitude of the PRCC is critical, since it indicates the strength of the effect the parameter has, regardless of sign. These methods have been used in studies for the spread of viral infection in order to elucidate trends about parameter dependence [42,43,44].

**Figure 7:**
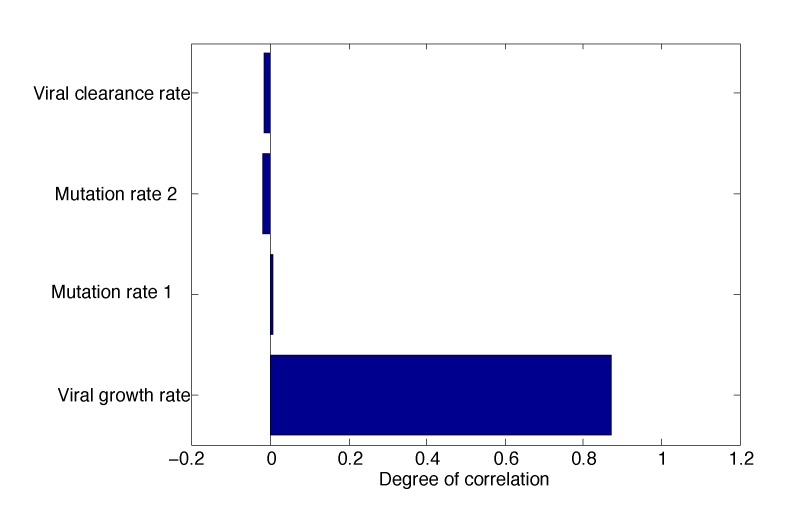
Tornado plot showing partial rank correlation coefficients (PRCCs) of *R*_0_ to its dependent parameters. The parameter with the largest impact on *R*_0_ is the viral growth rate, *r_W_*.

**Figure 8:**
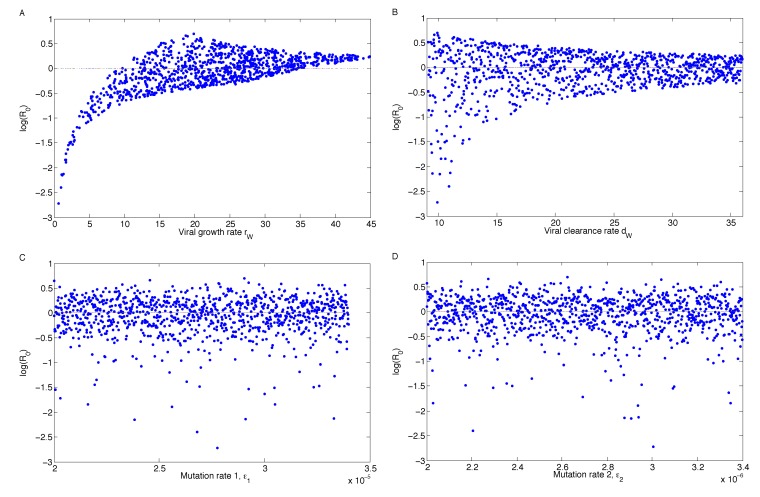
Landscape plots of *R*_0_ *vs*. *r_W_*, *d_W_*, *ϵ*_1_ and *ϵ*_2_ using Monte Carlo simulations. If the viral growth rate can be reduced below 10, then eradication is likely, regardless of variations in the other parameters.

As can be seen in Figure 7, the virus growth rate has the greatest impact on the outcome. The outcome here is the value of *R*_0_, which is the difference between eradication and persistence. The Monte Carlo simulations are illustrated in Figure 8. Each dot is one of 1000 simulations. The mutation rates have very little effect on *R*_0_ when they are varied, but the virus growth and clearance rates have a noticeable trend. 

Note that the *R*_0_ values for *r_W_* peak around *r_W_* = 20 and are thus higher than if the growth rate was maximized (e.g., *r_W_* = 45). This is because the viral growth and clearance rates are linked (see Appendix A), so if *r_W_* reaches its extreme, then the viral clearance rate has to be adjusted accordingly. The result is a narrowing of the range of *R*_0_ values.

**Figure 9:**
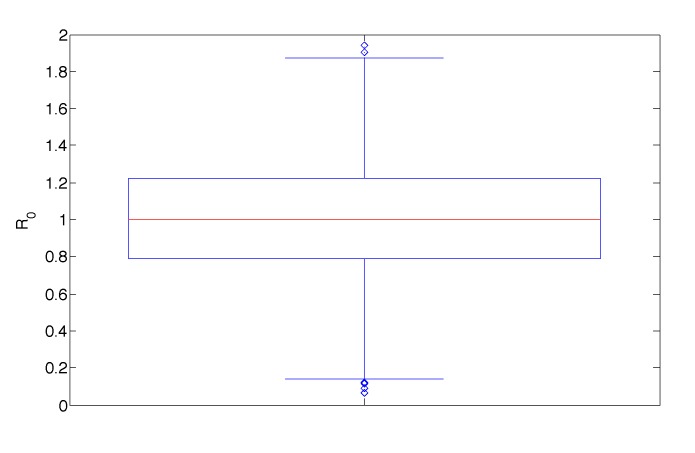
Box plot of distribution of *R*_0_ values as all parameters are varied throughout their ranges in Table 1 with *m* = 1. The median value of *R*_0_ is almost exactly one, suggesting that small variations in parameters can result in either viral persistence or eradication.

Finally, the box plot of variations in *R*_0_ as all parameters are varied is illustrated in Figure 9. This illustrates the median, interquartile range and extreme values of *R*_0_ across all parameter ranges. The median value is almost exactly one.

## 4. Discussion

In this study, we investigated virus clearance and escape with passive immunization, given various virus and neutralizing antibody characteristics. We showed that the eradication of all virus strains is possible with three infusions when the antibody infusion and neutralization rate against all strains are high enough (Figure 3B,C and Figure 4C). Virus persistence, in contrast, could occur when both mutants have resistance to antibody neutralization and a mutant strain has a high enough replication rate that allows it to escape after the antibodies decay (Figure 5B,C); when a mutant strain has strong enough antibody resistance (Figure 4B); or when a highly resistant, but low replicating, strain emerges easily, allowing the escape of a more moderately resistant, faster replicating strain (Figure 6). Furthermore, coexistence of multiple strains occurred when the antibody infusion and neutralization rate are low (Figure 3A, Figure 4A and Figure 5A); when the antibody resistance is high enough (Figure 5B); or when mutation rate *ϵ*_2_ is low enough and the Mutant 2 viral growth rate is in a specified range (Figure 1). See Table 2 for a summary.

Mathematically, we identified the steady states (*i.e.*, the long-term dynamics after the antibody has decayed) of the model; we calculated the basic reproduction number, *R*_0_; and we demonstrated the stability of the steady states, giving the regions of long-term viral persistence, for ranges of values of mutation rate *ϵ*_2_ and the growth rate of Mutant 2. Uncertainty and sensitivity analyses showed that the model parameter with the greatest effect on the outcome was the viral growth rate, *r_W_* (Figure 7 and Figure 8), which can lower *R*_0_ below one and eradicate the virus if it is sufficiently low.

Several features distinguish this study from previous work in this area. We used an impulsive differential equation model to investigate the scenarios that can theoretically give rise to the outcomes observed in viral infections with antibody vaccination. The model was parameterized using clinical data from the passive immunization of horses. The horses were given a pre-vaccination infusion and two post-infection infusions; correspondingly, our model included an initial antibody impulse before infection and two post-infection antibody impulses. In the horses, the passively transferred neutralizing antibody immunizations controlled all virus in some cases and in other cases allowed escape of a mutant strain. Accordingly, our model determined under which conditions the impulses could eradicate infection or could result in mutant escape.

This study shows the effects of magnification of the antibody effect. Magnification of this effect would be achieved by infusing more antibodies per impulse and increasing their neutralization ability. This illustrates the range of effectiveness of different vaccination strategies. If we had a vaccine that was more effective than the baseline vaccine, then different outcomes could occur, including mutant escape. Without this factor, the outcome is that the wild-type virus dominates, with both mutants present. If the magnification factor is increased moderately, then one or the other mutant could escape or we could have viral elimination. This gestures towards strategies for designing future vaccines: they will need to contain more antibodies and be more effective at neutralizing the virus; despite this, such vaccines run the risk of facilitating mutant escape.

Our model can compare the results found when mutants are equally susceptible to antibody neutral- ization with the results found when mutants are differentially susceptible to antibody neutralization. The model can also examine the effects of mutants with similar or vastly different mutation rates. Finally, our modeling results suggest how the escape of a mutant strain can emerge in the presence of a second, weaker mutant strain.

In addition, we provided calculations of kinetic parameters, including one (*p*) that previously had not been determined. We calculated the virus neutralization rate by antibodies from the initial amount of antibody infusion, the half-life of virus due to neutralizing antibody and the antibody decay rate (Appendix A and Table 1). We also calculated the overall growth rates of wild-type and mutant virus strains (Table 1) by fitting data from EIAV-infected SCID horses to our model, as in other studies [18,45]. See Appendix A.

It should be noted that, mathematically, a finite number of impulses cannot alter the stability properties of an equilibrium. In particular, no impulsive periodic orbit can be maintained; the system essentially restarts from the initial conditions resulting from the final impulse. However, we have shown that the result of these impulses can be to reduce the virus below the threshold of one virus particle in the horse. Our results show that four different outcomes are possible depending on the strain replication rate (*r*), the strain sensitivity to antibody neutralization (*p*) and the antibody magnification (*m*): eradication of both strains, Mutant 1 escape, Mutant 2 escape, or coexistence of both mutant strains. These indicate the conditions that can give rise to the mutant escape observed in the infected SCID horses [10,11]. With high antibody magnification, all strains are eradicated quickly with equal antibody neutralization rates, as well as with reduced antibody neutralization rates. However, Mutant 1 can escape when both mutant strains have 100-fold resistance. We note that, consistent with the results seen in the EIAV-specific antibody-infused infected SCID horses [10], the model shows no steady state with the persistence of only the wild-type strain. One of the horses studied by Taylor *et al.* [11] was given a low dose of purified antibody, and the emergent virus was 93% wild-type and 7% Mutant 1, which is consistent with our results when *m* = 1. Another horse was given heterologous challenge using an EIAV molecular clone; protection from infection did not occur. Unfortunately, however, the emergent virus could not be sequenced.

To develop an effective neutralizing antibody-eliciting vaccine, it is essential to have an understanding of the conditions that allow virus persistence in the face of neutralizing antibodies. The studies by Taylor *et al.* [11], which our model depicts, showed the blocking of lentiviral infection by a vaccine. The SCID horse with passive immunization provides a useful opportunity to witness the protective effects of three infusions of a defined antibody pool, independent of the contribution of a continually generated *in vivo* antibody response. Furthermore, our results could be relevant for individuals (including immunodeficient individuals) who do not (or cannot) make sufficient neutralizing antibody responses.

Our results rely upon a number of assumptions, which should be noted. We assume that different mutants have reduced growth rates, that viral clearance rates are the same for all viral strains and that the neutralizing antibody exerts its effects by reducing the virus population. We considered the scenario where mutation is continual; *i.e.*, mutant strains continually arise. We do not consider sequential mutation or reversion (*i.e.*, back mutation). The carrying capacities for each strain are different, and, to a large degree, our model results depend upon the differences between these carrying capacities. This is because the effective (scaled) carrying capacities depend on the growth rates of each viral strain. Furthermore, increasing the amount of the neutralizing antibody infusion may not be possible due to physical constraints, and increasing the neutralization rate (as well as preventing the emergence of resistance) may be difficult. The functional responses of antibody-dependent cell-mediated cytotoxicity (ADCC) and antibody-dependent cell-mediated viral inhibition (ADCVI) have not been included in our model, as it has been reported that ADCC is not necessary for the control of EIAV [46], and no studies to date have shown that ADCVI is involved in EIAV infection.

This study adds to our understanding of how antibodies control a viral infection of wild-type and two mutant strains. It gives insight into the development of vaccines and, specifically, what vaccine characteristics are needed for virus control. It indicates how high the antibody infusions need to be and how much neutralization is needed, theoretically, in order to prevent viral escape. The model shows which conditions can lead to the control of all strains, which conditions lead to the escape of all mutant strains, and which conditions lead to control of some strains but escape of others. This indicates the importance of the dosage and neutralization rate needed by passive immunization to control infection. These results may be applicable to other passively administered antibody therapies or potentially other passively transferred therapies (e.g., CTLs).

## 5. Conclusions

In conclusion, the results drawn from this study have important implications for viral control that can be used to guide vaccination strategies. In the studies by Taylor *et al.*, the infusion of broadly neutralizing antibodies (before infection and for two weeks following infection) protected some horses from EIAV infection, and other horses from wild-type EIAV infection but not from a neutralization-resistant EIAV variant [10,11]. The current study indicates which conditions (in terms of strain replication rate, antibody neutralization rate and amount of antibody) can theoretically give rise to these two outcomes, as well as others. Furthermore, our results quantify (on a relative scale) the amount of antibody infusion and antibody neutralization needed to block infection. A high antibody magnification (that affects the antibody neutralization rate, *p*, and the amount of infusions, *A^i^* and *A*_0_), resulting in eradication of wild-type and mutant strains, is important for developing an effective vaccine.

## Appendix

### A. Calculating Parameters

#### A.1. Calculating *p*

Consider a simplified version of the model without impulses or viral replication:

$$\begin{array}{l} V_{W}^{\prime }=-p_{W}V_{W}A\\ A\prime =-qA \end{array}$$

Then we have:

*A*(*t*) = *A*_0_*e**^−^**^q^**^t^*

$$V_{W}^{\prime }=-p_{W}A_{0}e^{-qt}V_{W}$$

$$\int_{0}^{t}\frac{V_{W}^{\prime }}{V_{W}}dt=-p_{W}A_{0}\int_{0}^{t}e^{-qt}$$

$$\text{ln}\frac{V_{W}}{V_{0}}=\frac{p_{W}A_{0}}{q}e^{-qt}-\frac{p_{W}A_{0}}{q}$$

$$V_{W}=V_{0}e^{-p_{W}A_{0}(1-e^{-qt})/q}$$

We know the half-life, *t*_1_*_/_*_2_, the time it takes for virus to be reduced to 50%. Thus:

$$\frac{V_{0}}{2}=V_{0}e^{-p_{W}A_{0}(1-e^{-qt_{1/2}})/q}$$

$$-\text{ln}2=-\frac{p_{W}A_{0}}{q}(1-e^{-qt_{1/2}})$$

$$p_{W}=\frac{q\ln2}{A_{0}(1{-}^{e-qt_{1/2}})}$$

This determines the neutralization rate in terms of quantifiable parameters *q*, *A*_0_ and *t*_1_*_/_*_2_.

#### A.2. Calculating *A*_0_

The parameter *A*_0_ was determined from the initial condition *A**_−_*_1_ by calculating the exponential decay after a single day had elapsed. Thus:

*A*_0_ = *A**_−_*_1_*e**^−^**^q^*

#### A.3. Calculating *r**_W_* and *K*

The wild-type net virus growth rate and virus carrying capacity were calculated by fitting the data from EIAV-infected control SCID horses (without EIAV-specific antibodies), A2245, A2247, H707 and H713 [10], to the differential equation for wild-type virus with antibody neutralization set to zero. After fitting the total growth rate, the clearance rate was subtracted to determine *r**_W_*. The virus growth rate for Mutant 1, *r**_M_*_1_, was determined equivalently by fitting data from infected EIAV-specific antibody-infused SCID horses, A2239 and A2240 [10], and subtracting the clearance rate.

To calculate the range of *r**_W_*, we allowed the difference (*r**_W_* − *d**_W_*) to range from −9.1 to 9.1 and then recovered *r* from the viral clearance rate in each Monte Carlo simulation. This is because the viral growth and clearance rates are linked, and the viral clearance rate ranges from 9.1 to 36 [38]. It follows that the practical outcome of this is that *r* ranges from zero to 46, but in such a way that *r**_W_* > *d**_W_*.

The carrying capacity in the absence of antibody neutralization was similarly fitted to EIAV-infected SCID horses and found to be *K̃* = 2.9 × 106 (range 1.9 × 106–9.7 × 106). These values were then scaled to $K=\tilde{K}(1-\frac{d_{W}}{r_{W}})$ to account for viral growth and clearance.

### B. The Non-Impulsive System

The non-impulsive system is equivalent to the impulsive model with *A**^i^* = 0.

#### B.1. Equilibria

If *A* ≠ 0 at equilibrium, then *p**_W_**V**_W_* + *p**_M_*_1_*V**_M_*_1_ + *p**_M_*_2_*V**_M_*_2_ = −*q* < 0. It follows that any equilibrium has *A* = 0.

If *V*_*W*_ = 0, then either *V*_*M*1_ = 0 or $r_{M1}(1-\frac{V}{K})=d_{M1}$. In the former case, either *V**_M_*_2_ = 0 or:

$$r_{M2}\left(1-\frac{V_{M2}}{K}\right)=d_{M2}$$

$$V_{M2}=K\left(1-\frac{d_{M2}}{r_{M2}}\right)$$

assuming $R_{2}=\frac{r_{M2}}{d_{M2}}>1$.

In the latter case,

$$r_{M2}V_{M2}\frac{d_{M1}}{r_{M1}}=d_{M2}V_{M2}$$

*V**_M_*_2_ = 0

Similarly,

$$r_{M1}\left(1-\frac{V_{M1}}{K}\right)=d_{M1}$$

$$V_{M1}=K\left(1-\frac{d_{M1}}{r_{M1}}\right)$$

assuming $R_{1}=\frac{r_{M1}}{d_{M1}}>1$ or *V_M_*_1_ = 0.

If *V**_W_* ≠ 0, then:

$$r_{W}\left(1-\frac{V}{K}\right)=\epsilon_{1}+\epsilon_{2}+d_{W}$$

$$r_{M1}V_{M1}\frac{\epsilon_{1}+\epsilon_{2}+d_{W}}{r_{W}}+\epsilon_{1}V_{W}=d_{M1}V_{M1}$$

$$V_{M1}=\frac{\epsilon_{1}r_{W}}{d_{M1}r_{W}-r_{M1}(\epsilon_{1}+\epsilon_{2}+d_{W})}V_{W}$$

assuming $R_{E1}=\frac{d_{M1}r_{W}}{r_{M1}(\epsilon_{1}+\epsilon_{2}+d_{W})}>1$.

$$r_{M2}V_{M2}\frac{\epsilon_{1}+\epsilon_{2}+d_{W}}{r_{W}}+\epsilon_{2}V_{W}=d_{M2}V_{M2}$$

$$V_{M2}=\frac{\epsilon_{2}r_{W}}{d_{M2}r_{W}-r_{M2}(\epsilon_{1}+\epsilon_{2}+d_{W})}V_{W}$$

assuming $R_{E2}=\frac{d_{M2}r_{W}}{r_{M2}(\epsilon_{1}+\epsilon_{2}+d_{W})}>1$.

Then *V**_W_* satisfies:

$${\overset{-}{V}}_{W}=\frac{K(r_{W}-\epsilon_{1}-\epsilon_{2}-d_{W})}{r_{W}\left(1+\frac{\epsilon_{1}r_{W}}{d_{M1}r_{W}-r_{M1}(\epsilon_{1}+\epsilon_{2}+d_{W})}+\frac{\epsilon_{2}r_{W}}{d_{M2}r_{W}-r_{M2}(\epsilon_{1}+\epsilon_{2}+d_{W})}\right)}$$

assuming $R_{E3}=\frac{r_{W}}{\epsilon_{1}+\epsilon_{2}+d_{W}}>1$.

Hence the equilibria of the non-impulsive system are:

$$(V_{W},V_{M1},V_{M2},A)=(0,0,0,0),(0,K\left(1-\frac{d_{M1}}{r_{M1}}\right),0,0),(0,0,K\left(1-\frac{d_{M2}}{r_{M2}}\right),0)\text{ and}$$

$$\left({\overset{-}{V}}_{W},\frac{\epsilon_{1}r_{W}}{d_{M1}r_{W}-r_{M1}(\epsilon_{1}+\epsilon_{2}+d_{W})}{\overset{-}{V}}_{W},\frac{\epsilon_{2}r_{W}}{d_{M2}r_{W}-r_{M2}(\epsilon_{1}+\epsilon_{2}+d_{W})}{\overset{-}{V}}_{W},0\right)$$

where *V_W_* is as above. These are, respectively, the disease-free equilibrium, the Mutant 1 equilibrium, the Mutant 2 equilibrium and the coexistence equilibrium.

Thus, if the wild-type exists at equilibrium, then so do both mutants. If there’s no wild-type at equilibrium, then one or the other mutant exists alone or not at all.

#### B.2. Jacobian

The Jacobian is *J* = [*J*_1_*|J*_2_], where:

$$J_{1}=\left[\begin{array}{cc} -p_{W}A+r_{W}\left(1-\frac{V}{K}\right)-\frac{r_{W}V_{W}}{K}-\epsilon_{1}-\epsilon_{2}-d_{W} & -\frac{r_{W}V_{W}}{K}\\ -\frac{r_{M1}V_{M1}}{K}+\epsilon_{1} & -p_{M1}A+r_{M1}\left(1-\frac{V}{K}\right)-\frac{r_{M1}V_{M1}}{K}-d_{M1}\\ -\frac{r_{M2}V_{M2}}{K}+\epsilon_{2} & -\frac{r_{M2}V_{M2}}{K}\\ -p_{W}A & -p_{M1}A \end{array}\right]$$

$$J_{2}=\left[\begin{array}{cc} -\frac{r_{W}V_{W}}{K} & -p_{W}V_{W}\\ -\frac{r_{M1}V_{M1}}{K} & -p_{M1}V_{M1}\\ -p_{M2}A+r_{M2}\left(1-\frac{V}{K}\right)-\frac{r_{M2}V_{M2}}{K}-d_{M2} & -p_{M2}V_{M2}\\ -p_{M2}A & -q-p_{W}V_{W}-p_{M1}V_{M1}-p_{M2}V_{M2} \end{array}\right]$$

We then have:

$$J{|}_{\;\;(0,0,0,0)}=\left[\begin{array}{cccc} r_{W}-\epsilon_{1}-\epsilon_{2}-d_{W} & 0 & 0 & 0\\ \epsilon_{1} & r_{M1}-d_{M1} & 0 & 0\\ \epsilon_{2} & 0 & r_{M2}-d_{M2} & 0\\ 0 & 0 & 0 & -q \end{array}\right]$$

This equilibrium is unstable if *R**_E_*_3_ > 1, or *R*_1_ > 1 or *R*_2_ > 1. Note that, for our parameter choices, *r**_j_**> d**_j_* (*j* = *W*, *M*1, *M*2), so *R*_1_ > 1 and *R*_2_ > 1. It follows that the disease-free equilibrium is always unstable.

Evaluating *J* at (0*, V**_M_*_1_, 0, 0), we have:

$$\left[\begin{array}{cccc} r_{W}\left(1-\frac{V_{M1}}{K}\right)-\epsilon_{1}-\epsilon_{2}-d_{W} & 0 & 0 & 0\\ -\frac{r_{M1}V_{M1}}{K}+\epsilon_{1} & r_{M1}\left(1-\frac{V_{M1}}{K}\right)-\frac{r_{M1}V_{M1}}{K}-d_{M1} & -\frac{r_{M1}V_{M1}}{K} & -p_{M1}V_{M1}\\ \epsilon_{2} & 0 & r_{M2}\left(1-\frac{V_{M1}}{K}\right)-d_{M2} & 0\\ 0 & 0 & 0 & -q-p_{M1}V_{M1} \end{array}\right]$$

The eigenvalues are:

$$\begin{array}{l} \lambda_{1,2,3,4}=r_{W}\left(1-\frac{V_{M1}}{K}\right)-\epsilon_{1}-\epsilon_{2}-d_{W},r_{M1}\left(1-\frac{V_{M1}}{K}\right)-\frac{r_{M1}V_{M1}}{K}-d_{M1},r_{M2}\left(1-\frac{V_{M1}}{K}\right)-d_{M2},-q-p_{M1}V_{M1}\\ =r_{W}\frac{d_{M1}}{r_{M1}}-\epsilon_{1}-\epsilon_{2}-d_{W},r_{M1}\frac{d_{M1}}{r_{M1}}-\frac{r_{M1}V_{M1}}{K}-d_{M1},r_{M2}\frac{d_{M1}}{r_{M1}}-d_{M2},-q-p_{M1}V_{M1}\\ =\frac{1}{r_{M1}}(d_{M1}r_{W}-r_{M1}(\epsilon_{1}+\epsilon_{2}+d_{W}),-\frac{r_{M1}V_{M1}}{K},\frac{d_{M1}r_{M2}-d_{M2}r_{M1}}{r_{M1}},-q-p_{M1}V_{M1} \end{array}$$

This equilibrium is unstable if *R**_E_*_1_ > 1 or if:

(1) *d**_M_*_1_*r**_M_*_2_− *d**_M_*_2_*r**_M_*_1_ > 0

(note that this condition usually fails in practice, since *d**_M_*_1_ = *d**_M_*_2_ and *r**_M_*_1_ > *r**_M_*_2_).

Finally, evaluating *J* at (0, 0, *V**_M_*_2_, 0), we have:

$$\left[\begin{array}{cccc} r_{W}\left(1-\frac{V_{M2}}{K}\right)-\epsilon_{1}-\epsilon_{2}-d_{W} & 0 & 0 & 0\\ \epsilon_{1} & r_{M1}\left(1-\frac{V_{M2}}{K}\right)-d_{M1} & 0 & 0\\ -\frac{r_{M2}V_{M2}}{K}+\epsilon_{2} & -\frac{r_{M2}V_{M2}}{K} & r_{M2}\left(1-\frac{V_{M2}}{K}\right)-\frac{r_{M1}V_{M1}}{K}-d_{M2} & -p_{M2}V_{M2}\\ 0 & 0 & 0 & -q-p_{M2}V_{M2} \end{array}\right]$$

The eigenvalues are:

$$\begin{array}{l} \lambda_{1,2,3,4}=r_{W}\left(1-\frac{V_{M2}}{K}\right)-\epsilon_{1}-\epsilon_{2}-d_{W},r_{M1}\left(1-\frac{V_{M2}}{K}\right)-d_{M1},\\ r_{M2}\left(1-\frac{V_{M2}}{K}\right)-\frac{r_{M2}V_{M2}}{K}-d_{M2},-q-p_{M2}V_{M2}\\ =r_{W}\frac{d_{M2}}{r_{M2}}-\epsilon_{1}-\epsilon_{2}-d_{W},r_{M1}\frac{d_{M2}}{r_{M2}}-d_{M1},r_{M2}\frac{d_{M2}}{r_{M2}}-\frac{r_{M2}V_{M2}}{K}-d_{M2},-q-p_{M2}V_{M2}\\ =\frac{1}{r_{M2}}(d_{M2}r_{W}-r_{M2}(\epsilon_{1}+\epsilon_{2}+d_{W}),\frac{d_{M2}r_{M1}-d_{M1}r_{M2}}{r_{M2}},-\frac{r_{M2}V_{M2}}{K},-q-p_{M2}V_{M2} \end{array}$$

This equilibrium is unstable if *R**_E_*_2_ > 1 or if Condition (1) fails. As noted above, Condition (1) usually fails in practice, suggesting that this equilibrium is usually unstable.

Note in particular that Condition (1) implies that the two mutant-only equilibria cannot both be stable.

#### B.3. Calculating *R*_0_

Using the existence of the endemic equilibrium method [47], the disease is endemic if:

max{*R*_1_, *R*_2_, *R**_E_*_1_, *R**_E_*_2_, *R**_E_*_3_*}* > 1

However, note that *R**_E_*_3_ > *R*_1_> *R*_2_ and *R**_E_*_3_ > *R**_E_*_2_ > *R**_E_*_1_. Thus, the condition for the disease to persist is:

*R*_0_ = *R**_E_*_3_ > 1

### C. Stabilizing the Mutant 1 Equilibrium

We can write *r**_W_* = *d**_W_* + *γ*, where *γ* ≈ 0.5. Then:

$$R_{E3}=\frac{d_{W}+\gamma }{\epsilon_{1}+\epsilon_{2}+d_{W}}>1$$

unless mutation is of the order of ≈ 0.5, which is unrealistic. Thus, *R**_E_*_3_ is likely to be greater than one in all realistic situations.

Next, we investigate the possibility of stabilizing the Mutant 1 equilibrium. First note that Condition (1) usually fails. It follows that the Mutant 1 equilibrium is stable (and the coexistence equilibrium does not exist) if *R**_E_*_1_ < 1.

Suppose *r**_M_*_1_ = *r**_W_* − 0.01. Then we can write:

$$R_{E1}=\frac{d_{M1}r_{W}}{\left(r_{W}-0.01)(\epsilon_{1}+\epsilon_{2}+d_{W}\right)}=1$$

(We choose 0.01, since this brings *r**_M_*_1_ extremely close to *r**_W_*. Larger variations will make this worse. If the value is negative, then this can never equal one.)

Solving, we have:

$$\epsilon_{1}+\epsilon_{2}=\frac{d_{M1}r_{W}}{r_{W}-0.01}-d_{W}=9.7997\times 10^{-3}$$

Thus, if $\epsilon_{1}+\epsilon_{2}$ is sufficiently large, it can stabilize the mutant-only equilibrium. In particular, if mutation for the second mutant is sufficiently large, it can stabilize the first mutant. This is a surprising result.

To be specific, this would be the case where there was extremely high mutation rate for the second mutant, but the first mutant still had a replication advantage (so *r*_*M*1_ > *r*_*M*2_, but $\epsilon_{2}$ is very large indeed). That is, the wild-type would mutate easily to a mutant that did not replicate very well.

If this does not happen, then both mutants are likely to persist. The system may stabilize at the interior equilibrium or it may potentially persist in some form, such as a periodic orbit or chaos.

## References

[1] Liu P., Overman R.G., Yates N.L., Alam S.M., Vandergrift N., Chen Y., Graw F., Freel S.A., Kappes J.C., Ochsenbauer C. (2011). Dynamic antibody specificities and virion concentrations in circulating immune complexes in acute to chronic HIV-1 infection. J. Virol..

[2] Tomaras G.D., Yates N.L., Liu P., Qin L., Fouda G.G., Chavez L.L., Decamp A.C., Parks R.J., Ashley V.C., Lucas J.T. (2008). Initial B-cell responses to transmitted human immunodeficiency virus type 1: Virion-binding immunoglobulin M (IgM) and IgG antibodies followed by plasma anti-gp41 antibodies with ineffective control of initial viremia. J. Virol..

[3] Leroux C., Cadore J.L., Montelaro R.C. (2004). Equine Infectious Anemia Virus (EIAV): What has HIV’s country cousin got to tell us?. Vet. Res..

[4] Maury W., Oaks J.L., Desport M. (2010). Equine infectious anemia virus pathogenesis and replication. Lentiviruses and Macrophages: Molecular and Cellular Interactions.

[5] Mealey R.H., Fraser D.G., Oaks J.L., Cantor G.H., McGuire T.C. (2001). Immune reconstitution prevents continuous equine infectious anemia virus replication in an Arabian foal with severe combined immunodeficiency: Lessons for control of lentiviruses. Clin. Immunol..

[6] Perryman L.E., O’Rourke K.I., McGuire T.C. (1988). Immune responses are required to terminate viremia in equine infectious anemia lentivirus infection. J. Virol..

[7] Carpenter S., Chen W.C., Dorman K.S. (2011). Rev variation during persistent lentivirus infection. Viruses.

[8] Mealey R.H., Leib S.R., Littke M.H., Wagner B., Horohov D.W., McGuire T.C. (2009). Viral load and clinical disease enhancement associated with a lentivirus cytotoxic T lymphocyte vaccine regimen. Vaccine.

[9] Wu W., Blythe D.C., Loyd H., Mealey R.H., Tallmadge R.L., Dorman K.S., Carpenter S. (2011). Decreased infectivity of a neutralization-resistant equine infectious anemia virus variant can be overcome by efficient cell-to-cell spread. J. Virol..

[10] Taylor S.D., Leib S.R., Carpenter S., Mealey R.H. (2010). Selection of a rare neutralization-resistant variant following passive transfer of convalescent immune plasma in equine infectious anemia virus-challenged SCID horses. J. Virol..

[11] Taylor S.D., Leib S.R., Wu W., Nelson R., Carpenter S., Mealey R.H. (2011). Protective effects of broadly neutralizing immunoglobulin against homologous and heterologous equine infectious anemia virus infection in horses with severe combined immunodeficiency. J. Virol..

[12] Mascola J.R., Lewis M.G., Stiegler G., Harris D., VanCott T.C., Hayes D., Louder M.K., Brown C.R., Sapan C.V., Frankel S.S. (1999). Protection of Macaques against pathogenic simian/human immunodeficiency virus 89.6PD by passive transfer of neutralizing antibodies. J. Virol..

[13] Ng C.T., Jaworski J.P., Jayaraman P., Sutton W.F., Delio P., Kuller L., Anderson D., Landucci G., Richardson B.A., Burton D.R. (2010). Passive neutralizing antibody controls SHIV viremia and enhances B cell responses in infant macaques. Nat. Med..

[14] Nishimura Y., Igarashi T., Haigwood N.L., Sadjadpour R., Donau O.K., Buckler C., Plishka R.J., Buckler-White A., Martin M.A. (2003). Transfer of neutralizing IgG to macaques 6 h but not 24 h after SHIV infection confers sterilizing protection: Implications for HIV-1 vaccine development. Proc. Natl. Acad. Sci. USA.

[15] Ruprecht R.M., Ferrantelli F., Kitabwalla M., Xu W., McClure H.M. (2009). Antibody protection: Passive immunization of neonates against oral AIDS virus challenge. Methods Mol. Biol..

[16] Shibata R., Igarashi T., Haigwood N., Buckler-White A., Ogert R., Ross W., Willey R., Cho M.W., Martin M.A. (1999). Neutralizing antibody directed against the HIV-1 envelope glycoprotein can completely block HIV-1/SIV chimeric virus infections of macaque monkeys. Nat. Med..

[17] Schwartz E.J., Pawelek K.A., Harrington K., Cangelosi R., Madrid S. (2013). Immune control of equine infectious anemia virus infection by CTLs and antibodies. Appl. Math..

[18] Ciupe S., Schwartz E.J. (2014). Understanding virus-host dynamics following EIAV infection in SCID horses. J. Theor. Biol..

[19] Smith? R.J., Schwartz E.J. (2008). Predicting the potential impact of a cytotoxic T-lymphocyte HIV vaccine: How often should you vaccinate and how strong should the vaccine be?. Math. Biosci..

[20] Konrad B.P., Vaidya N.K., Smith? R.J. (2011). Modelling mutation to a cytotoxic T-lymphocyte HIV vaccine. Math. Pop. Stud..

[21] Bainov D.D., Simeonov P.S. (1989). Systems with Impulsive Effect.

[22] Bainov D.D., Simeonov P.S. (1993). Impulsive differential equations: Periodic solutions and applications.

[23] Bainov D.D., Simeonov P.S. (1995). Impulsive Differential Equations: Asymptotic Properties of the Solutions.

[24] Lakshmikantham V., Bainov D.D., Simeonov P.S. (1989). Theory of Impulsive Differential Equations.

[25] Agur Z., Cojocaru L., Mazor G., Anderson R., Danon Y. (1993). Pulse mass measles vaccination across age cohorts. Proc. Natl. Acad. Sci. USA.

[26] Stone L., Olinky R., Huppert A. (2007). Seasonal dynamics of recurrent epidemics. Nature.

[27] Miron R.E., Smith? R.J. (2010). Modelling imperfect adherence to HIV induction therapy. BMC Infect. Dis..

[28] Roberts M.G., Kao R.R. (1998). The dynamics of an infectious disease in a population with birth pulses. Math. Biosci..

[29] Al-arydah M., Smith? R.J., Lutscher F. (2012). Modelling gender-structured wildlife diseases with harvesting: Chronic Wasting Disease as an example. ISRN Biomath..

[30] Kot M. (2001). Elements of Mathematical Ecology.

[31] Schwartz E.J., Neumann A.U., Teixeira A.V., Bruggeman L.A., Rappaport J., Perelson A.S., Klotman P.E. (2002). Effect of target cell availability on HIV-1 production *in vitro*. AIDS.

[32] Waldmann T.A., Strober W. (1969). Metabolism of immunoglobulins. Prog. Allergy.

[33] Metzner K.J., Jin X., Lee F.V., Gettie A., Bauer D.E., di Mascio M., Perelson A.S., Marx P.A., Ho D.D., Kostrikis L.G. (2000). Effects of *in vivo* CD8^+^ T cell depletion on virus replication in rhesus macaques immunized with a live, attenuated simian immunodeficiency virus vaccine. J. Exp. Med..

[34] Nowak M.A., Lloyd A.L., Vasquez G.M., Wiltrout T.A., Wahl L.M., Bischofberger N., Williams J., Kinter A., Fauci A.S., Hirsch V.M. (1997). Viral dynamics of primary viremia and antiretroviral therapy in simian immunodeficiency virus infection. J. Virol..

[35] Van Rompay K.K., Singh R.P., Pahar B., Sodora D.L., Wingfield C., Lawson J.R., Marthas M.L., Bischofberger N. (2004). CD8^+^-cell-mediated suppression of virulent simian immunod- eficiency virus during tenofovir treatment. J. Virol..

[36] Mansky L.M., Temin H.M. (1995). Lower *in vivo* mutation rate of human immunodeficiency virus type 1 than that predicted from the fidelity of purified reverse transcriptase. J. Virol..

[37] Bryan W.R. (1957). Interpretation of host response in quantitative studies on animal viruses. Ann. NY Acad. Sci..

[38] Ramratnam B., Bonhoeffer S., Binley J., Hurley A., Zhang L., Mittler J.E., Markowitz M., Moore J.P., Perelson A.S., Ho D.D. (1999). Rapid production and clearance of HIV-1 and hepatitis C virus assessed by large volume plasma apheresis. Lancet.

[39] Heffernan J.M., Smith R.J., Wahl L.M. (2005). Perspectives on the basic reproductive ratio. J. R. Soc. Interface.

[40] Schwartz E.J., Vaidya N.K., Dorman K., Carpenter S., Mealey R.H. (2014). Dynamics of lentiviral
infection *in vivo* in the absence of adaptive host immune responses. J. Virol..

[41] Blower S.M., Dowlatabadi H. (1994). Sensitivity and uncertainty analysis of complex models of disease transmission: An HIV model, as an example. Int. Stat. Rev..

[42] Schwartz E.J., Blower S. (2005). Predicting the potential individual- and population-level effects of imperfect herpes simplex virus type 2 vaccines. J. Infect. Dis..

[43] Schwartz E.J., Bodine E.N., Blower S. (2007). Effectiveness and efficiency of imperfect therapeutic HSV-2 vaccines. Hum. Vaccines.

[44] Smith? R.J., Cloutier P., Harrison J., Desforges A., Mushayabasa S., Bhunu C.P. (2012). A mathematical model for the eradication of Guinea Worm Disease. Understanding the Dynamics of Emerging and Re-Emerging Infectious Diseases Using Mathematical Models.

[45] Schwartz E.J., Nanda S. (2014). Antibody Escape Kinetics of EIAV Infection of Horses. J. Virol..

[46] Tschetter J.R., Byrne K.M., Perryman L.E., McGuire T.C. (1997). Control of equine infectious anemia virus is not dependent on ADCC mediating antibodies. Virology.

[47] Li J., Blakeley D., Smith? R.J. (2011). The Failure of *R*_0_. Comp. Math. Methods Med..

